# *DERL3* Predicts and Drives Acquired Sunitinib Resistance in Renal Cell Carcinoma by Suppressing ER Stress–ROS-Dependent Apoptosis

**DOI:** 10.3390/biomedicines14081841

**Published:** 2026-08-15

**Authors:** Zhishu Zhang, Sihan Zhang, Peihua Wang, Degang Ding, Yuanxiang Lu, Xudong Zhu

**Affiliations:** 1 Department of Urology, Zhengzhou University People’s Hospital, Henan Provincial People’s Hospital, Zhengzhou 450003, China; 2 Department of Breast Surgery, Zhengzhou University People’s Hospital, Henan Provincial People’s Hospital, Zhengzhou 450003, China; 3 College of Veterinary Medicine, Henan Agricultural University, Zhengzhou 450002, China

**Keywords:** ccRCC, sunitinib resistance, *DERL3*, endoplasmic reticulum stress, ERAD, ROS, apoptosis, VEGFR-TKI

## Abstract

**Background**: Sunitinib remains an important VEGFR-targeted therapy for advanced clear cell renal cell carcinoma (ccRCC), but acquired resistance frequently limits its clinical benefit. The molecular mechanisms underlying sunitinib resistance remain insufficiently understood. **Methods**: Sunitinib-resistant ccRCC models were generated by serial in vivo selection using Caki-1 and 786-O xenografts. Transcriptomic profiling, integration with GSE76068, and siRNA-based screening were performed to identify candidate resistance drivers. *DERL3* expression and function were validated using qRT-PCR, Western blot, immunohistochemistry, gain- and loss-of-function assays, xenograft models, and mechanistic analyses of endoplasmic reticulum stress, ROS, and apoptosis. **Results**: Serial in vivo selection established stable sunitinib-resistant Caki-1-SR and 786-O-SR cells with markedly increased IC50 values. Integrated transcriptomic analysis identified six consistently upregulated genes in resistant models and GSE76068, among which *DERL3* knockdown most strongly restored sunitinib sensitivity. *DERL3* was upregulated in resistant ccRCC cells and clinical resistant specimens. High intratumoral *DERL3* expression was associated with poor response to neoadjuvant sunitinib and shorter progression-free and overall survival. Functionally, *DERL3* overexpression increased sunitinib resistance in vitro and in vivo, whereas *DERL3* silencing restored drug sensitivity. Mechanistically, *DERL3* depletion activated pro-apoptotic endoplasmic reticulum stress, increased ROS accumulation, and enhanced caspase-dependent apoptosis. Suppression of endoplasmic reticulum stress reduced ROS generation and apoptosis induced by *DERL3* knockdown. In resistant xenografts, *DERL3*-targeted inhibition enhanced the antitumor efficacy of sunitinib. **Conclusions**: *DERL3* is a clinically relevant driver of acquired sunitinib resistance in ccRCC. Targeting *DERL3* may restore sunitinib sensitivity by reactivating pro-apoptotic endoplasmic reticulum stress and ROS-dependent apoptosis.

## 1. Introduction

Renal cell carcinoma (RCC) is one of the most common malignancies of the urinary system, with clear cell RCC (ccRCC) representing the predominant histological subtype [1,2]. Approximately 25–30% of patients present with metastatic or unresectable disease at initial diagnosis, and an additional 20–40% of patients treated with curative-intent surgery subsequently develop recurrent and/or metastatic disease; many of these patients ultimately require systemic therapy [3,4,5,6,7]. Over the past two decades, treatment strategies for advanced ccRCC have evolved from cytokine-based therapy to vascular endothelial growth factor/vascular endothelial growth factor receptor (VEGF/VEGFR)-targeted tyrosine kinase inhibitors and, more recently, immune checkpoint inhibitor-based combinations [8,9]. Despite these advances, therapeutic resistance remains a major cause of treatment failure and poor survival.

Sunitinib is an oral multitargeted tyrosine kinase inhibitor that inhibits VEGFRs, platelet-derived growth factor receptors (PDGFRs), KIT, FLT3, and related kinases [10]. In the pivotal phase III trial, sunitinib significantly prolonged progression-free survival compared with interferon-α in previously untreated metastatic ccRCC, establishing VEGFR-targeted therapy as a major therapeutic strategy in ccRCC [11,12]. Although immune checkpoint inhibitor (ICI)-based combinations, including ICI plus TKI and dual checkpoint blockade, are now preferred first-line options for many patients with advanced ccRCC, sunitinib remains clinically relevant as a comparator in landmark trials, an alternative when ICI therapy is contraindicated or unavailable, and a model agent for studying VEGFR-TKI resistance [9,13,14,15]. However, primary resistance and acquired resistance to sunitinib are frequently observed. Tumors that initially respond often resume growth under continued VEGFR blockade, indicating that adaptive survival programs can bypass antiangiogenic and direct antitumor pressure [16,17].

Primary and acquired resistance to sunitinib are multifactorial. Reported mechanisms include activation of bypass angiogenic and growth signaling, epithelial–mesenchymal transition, lysosomal sequestration and reduced intracellular drug availability, metabolic and redox adaptation, epigenetic reprogramming, altered cell death thresholds, and remodeling of the tumor microenvironment [17,18,19,20,21,22,23,24]. Recent ccRCC studies further show that sunitinib can select ferroptosis-resistant cell states, activate a GCN2-ATF4-dependent serine synthesis program, and sustain HIF2α signaling through altered ubiquitin-dependent protein turnover [22,23,24]. These observations underscore the heterogeneity of resistance and the limited availability of clinically validated biomarkers. Therefore, identifying robust molecular drivers that are conserved between experimental resistance models and clinically derived datasets is essential for developing rational combination strategies.

Endoplasmic reticulum-associated degradation (ERAD) and the unfolded protein response (UPR) are central adaptive mechanisms that maintain proteostasis under cellular stress [25,26,27]. When misfolded or unfolded proteins accumulate in the endoplasmic reticulum, the UPR is activated to restore homeostasis by reducing protein synthesis, increasing chaperone expression, and enhancing ERAD [25,27]. Cancer cells frequently exploit ER stress adaptation to survive hypoxia, nutrient deprivation, oxidative stress, and drug exposure [28,29]. Therefore, ER proteostasis regulators may represent important mediators of therapeutic resistance. The mammalian Derlin family comprises *DERL1*, *DERL2*, and *DERL3*, integral endoplasmic reticulum membrane proteins that cooperate with ERAD complexes to recognize and retrotranslocate selected misfolded proteins for proteasomal degradation [30,31,32,33]. *DERL2* and *DERL3* are UPR-inducible, and *DERL3* can remodel ERAD complex assembly during sustained ER stress [30,32]. Cancer cells may exploit this quality-control machinery to survive hypoxia, nutrient deprivation, oxidative stress, and drug exposure [28,29,34,35,36]. In RCC, experimentally increasing ER stress has also been linked to restored sunitinib sensitivity [37]. These biological features, together with the cross-dataset and functional screening strategy described above, provided the rationale for prioritizing *DERL3* rather than selecting it solely from prior tumor-association studies [38]. Whether *DERL3* directly contributes to acquired sunitinib resistance in ccRCC remains unknown.

In this study, we established stable sunitinib-resistant ccRCC models through serial in vivo drug selection, integrated our transcriptomic data with the GSE76068 sunitinib-resistant ccRCC patient-derived xenograft dataset, and identified *DERL3* as a candidate resistance driver. We further evaluated its clinical significance in neoadjuvant sunitinib-treated ccRCC specimens and explored the mechanistic link between *DERL3*, ER stress, ROS accumulation, apoptosis, and sunitinib sensitivity.

## 2. Materials and Methods

### 2.1. Study Design

This study aimed to identify key molecular determinants of acquired sunitinib resistance in clear cell renal cell carcinoma (ccRCC) and to determine whether *DERL3* contributes to resistance through regulation of endoplasmic reticulum-associated degradation, ER stress, ROS accumulation, and apoptosis. We conducted a translational experimental study integrating in vivo-selected sunitinib-resistant ccRCC models, RNA sequencing, public dataset validation, siRNA-based functional screening, gain- and loss-of-function assays, mechanistic analyses, and xenograft experiments. In parallel, a retrospective clinical validation cohort was established using archived tumor specimens from patients with histologically confirmed ccRCC who received preoperative sunitinib and subsequent surgical resection at Henan Provincial People’s Hospital from 2020 to 2022. Clinical response was assessed using radiographic criteria, and *DERL3* expression was evaluated by immunohistochemistry.

### 2.2. Cell Culture

Human ccRCC cell lines Caki-1 and 786-O were obtained from the American Type Culture Collection (ATCC, Manassas, VA, USA). According to ATCC recommendations and previously published ccRCC cell culture protocols, Caki-1 cells were maintained in McCoy’s 5A medium supplemented with 10% fetal bovine serum (FBS), whereas 786-O cells were maintained in RPMI-1640 medium supplemented with 10% FBS. Both media were additionally supplemented with 100 U/mL penicillin and 100 μg/mL streptomycin. Cells were cultured at 37 °C in a humidified atmosphere containing 5% CO_2_ and passaged at approximately 70–80% confluence using 0.25% trypsin–EDTA. All experiments were performed using cells in the logarithmic growth phase. Cell lines were authenticated by short tandem repeat profiling and routinely tested negative for mycoplasma contamination before use.

### 2.3. Establishment of In Vivo-Selected Sunitinib-Resistant ccRCC Cell Lines

To generate acquired sunitinib-resistant ccRCC models that better recapitulate in vivo drug pressure, Caki-1 and 786-O cells were subcutaneously injected into the flanks of male BALB/c nude mice. When tumors became palpable or reached approximately 80 mm^3^, mice received sunitinib by oral gavage at 40 mg/kg/day. After approximately 4 weeks of treatment, residual tumors were harvested under sterile conditions. Tumor tissues were mechanically and enzymatically dissociated to obtain viable tumor cells, which were briefly expanded in vitro and then re-injected into a new cohort of nude mice. The same sunitinib selection procedure was repeated for three consecutive rounds. The final resistant derivatives were designated Caki-1-SR and 786-O-SR, respectively. As a sensitive control, parental cells were isolated from tumors of mice treated with vehicle (PBS, 10 mL/kg, p.o., daily), without any sunitinib selection.

### 2.4. Cell Viability and IC50 Assay

Cell viability and sunitinib sensitivity were assessed using the Cell Counting Kit-8 (CCK-8) assay (Dojindo Laboratories, Kumamoto, Japan). Briefly, Caki-1, 786-O, Caki-1-SR, and 786-O-SR cells were seeded into 96-well plates at a density of 4 × 10^3^ cells per well in 100 μL of complete medium and allowed to adhere overnight. Cells were then treated with increasing concentrations of sunitinib or an equivalent volume of dimethyl sulfoxide (DMSO) as the vehicle control for 72 h. After treatment, 10 μL of CCK-8 reagent was added to each well and incubated at 37 °C for 1.5–2 h. The absorbance was measured at 450 nm using a microplate reader. Cell viability was calculated as follows: $Cell\;viability\;(\%)=[\frac{{OD}_{treatment}-{OD}_{blank}}{{OD}_{vehicle}-{OD}_{blank}}]\times 100$. Dose-response curves were generated, and IC50 values were calculated using nonlinear regression analysis in GraphPad Prism (Version 10.2.0 (335)). All experiments were performed with at least three technical replicates per condition and repeated independently three times.

### 2.5. RNA Sequencing and Bioinformatic Analysis

Total RNA was extracted from parental and sunitinib-resistant ccRCC cells using TRIzol reagent according to the manufacturer’s instructions. RNA concentration and purity were assessed using a NanoDrop 2000 spectrophotometer (Thermo Fisher Scientific, Waltham, MA, USA), and RNA integrity was evaluated using an Agilent 2100 Bioanalyzer (Agilent Technologies, Inc., Santa Clara, CA, USA). Samples with an RNA integrity number greater than 7.0 were used for library construction. RNA sequencing was performed with three independent biological replicates per group. Poly(A)+ mRNA was enriched from total RNA using oligo(dT) magnetic beads, fragmented, and reverse-transcribed into cDNA. Sequencing libraries were generated after end repair, adaptor ligation, and PCR amplification, and paired-end 150-bp sequencing was performed on an Illumina NovaSeq 6000 platform (Illumina, Inc., San Diego, CA, USA).

Raw paired-end reads were processed with fastp (version 0.23.2) using the same filtering and adapter-trimming settings for every sample. Adapter-contaminated reads, low-quality reads, and reads containing excessive ambiguous bases were removed. Clean reads were aligned to the human GRCh38/hg38 reference genome with HISAT2, and gene-level counts were generated with featureCounts (Version 2.0.1) using the corresponding gene annotation. Genes with zero counts across all samples were excluded before differential analysis. DESeq2 was used for median-of-ratios size factor normalization and Wald testing, and multiple comparisons were controlled by the Benjamini–Hochberg method. Genes with an adjusted *p* value < 0.05 and an absolute log2 fold change > 1 were considered differentially expressed. All steps were performed on three independent biological replicates per group using an identical analysis workflow.

Overlapping candidates were visualized using volcano plots, and heatmaps. Gene identifiers were mapped consistently before enrichment analysis. Over-representation analysis for Gene Ontology and Kyoto Encyclopedia of Genes and Genomes pathways was performed with clusterProfiler in R, with Benjamini–Hochberg adjustment. Gene set enrichment analysis used genes ranked by the signed DESeq2 test statistic; normalized enrichment scores and false-discovery-rate-adjusted *p* values were reported. An adjusted *p* value < 0.05 was considered significant.

### 2.6. siRNA Screening of Candidate Resistance Genes

Six differentially expressed genes identified by integrated transcriptomic analysis, including *DERL3*, *ZDHHC2*, *SNORA33*, *NPRL2*, *TRIB3*, and *CD276*, were selected for functional screening. Gene-specific siRNAs and a non-targeting control siRNA were synthesized by GenePharma Co., Ltd. (Shanghai, China), and the sequences are listed in Supplementary Table S1. Cells were transfected with siRNAs at a final concentration of 50 nM using Lipofectamine RNAiMAX reagent (Invitrogen, Thermo Fisher Scientific, Waltham, MA, USA) according to the manufacturer’s instructions. Knockdown efficiency was assessed 48 h after transfection by qRT-PCR and, when applicable, by Western blot.

For drug sensitivity screening, siRNA-transfected cells were treated with increasing concentrations of sunitinib 24 h after transfection. Cell viability was measured after 72 h using the CCK-8 assay, and IC50 values were calculated by nonlinear regression analysis in GraphPad Prism (Version 10.2.0 (335)). The gene whose knockdown produced the greatest reduction in sunitinib IC50 was prioritized for further validation. All experiments were independently repeated at least three times.

### 2.7. qRT-PCR

Total RNA was isolated using TRIzol (Invitrogen, Carlsbad, CA, USA) reagent and reverse-transcribed into cDNA using the PrimeScript RT reagent Kit with gDNA Eraser (Takara, Kusatsu, Shiga, Japan). qRT-PCR was performed using TB Green Premix Ex Taq II (Takara, Kusatsu, Shiga, Japan) on an Applied Biosystems 7500 Real-Time PCR System (Applied Biosystems, Foster City, CA, USA). The reaction conditions were 95 °C for 30 s, followed by 40 cycles of 95 °C for 5 s and 60 °C for 34 s. GAPDH was used as the endogenous control, and relative gene expression was calculated using the 2^−ΔΔCt^ method. Each sample was analyzed in triplicate, and the experiment was independently repeated three times. Primer sequences are provided in Supplementary Table S2.

### 2.8. Western Blot

Cells or tumor tissues were lysed in RIPA buffer supplemented with protease and phosphatase inhibitors. Equal amounts of protein were separated by SDS-PAGE and transferred onto PVDF membranes. Membranes were blocked and incubated with primary antibodies against *DERL3*, *DDIT3*, *CASP3*, *CASP6*, *BAX*, and *β-actin*, followed by HRP-conjugated secondary antibodies and enhanced chemiluminescence detection. Antibody specificity was evaluated by detection at the expected molecular mass and by treatment- or genotype-consistent signal changes. In-study validation of the anti-*DERL3* antibody was additionally supported by reciprocal signal gain after *DERL3* overexpression and signal loss after two independent *DERL3*-targeting shRNAs. Non-saturated exposures were used for densitometry. Target-band intensity was normalized to β-actin, and the matched control was set to 1.

### 2.9. Plasmid Overexpression and shRNA-Mediated Knockdown

For gain-of-function experiments, parental Caki-1 and 786-O cells were transduced or transfected with *DERL3*-overexpression vectors or empty vector controls. Stable cells were selected using puromycin when applicable. For loss-of-function experiments, Caki-1-SR and 786-O-SR cells were transduced with two independent shRNAs targeting *DERL3* or a non-targeting shRNA control. Knockdown or overexpression efficiency was validated by qRT-PCR and Western blot.

### 2.10. Clinical Specimens and Immunohistochemistry

ccRCC tumor specimens were retrospectively collected from patients who received neoadjuvant sunitinib followed by surgical resection at Henan Provincial People’s Hospital between 2020 and 2022. Eligible patients had histologically confirmed ccRCC, available formalin-fixed paraffin-embedded tumor tissue, complete clinicopathological data, radiographic response assessment, and follow-up information. Patients with insufficient tissue, incomplete treatment records, or missing follow-up data were excluded. Treatment response was evaluated according to RECIST 1.1 criteria using contrast-enhanced CT or MRI before and after neoadjuvant sunitinib. Patients with partial response (PR) were classified as the sensitive group, whereas those with progressive disease (PD) or stable disease (SD) without meaningful tumor shrinkage were classified as the resistant group.

Immunohistochemistry was performed on formalin-fixed, paraffin-embedded tumor sections. Sections were deparaffinized, rehydrated, subjected to antigen retrieval, blocked for endogenous peroxidase activity, and incubated with an anti-*DERL3* antibody overnight at 4 °C. Staining was visualized with DAB and counterstained with hematoxylin. *DERL3* immunohistochemical expression was quantified as the percentage of positively stained tumor cells across each whole section, yielding a value from 0% to 100%. Two pathologists independently quantified all slides while blinded to treatment response, survival outcomes, and molecular results.

### 2.11. ROS Detection

Intracellular ROS levels were detected using a DCFH-DA probe (Sigma-Aldrich, St. Louis, MO, USA). Briefly, cells were incubated with 10 μM DCFH-DA in serum-free medium at 37 °C for 30 min in the dark, washed three times with PBS, and analyzed by flow cytometry using the FITC channel. Mean fluorescence intensity was used to quantify intracellular ROS levels. For ER stress inhibition experiments, cells were pretreated with 2 mM 4-phenylbutyric acid (4-PBA) for 2 h before sunitinib treatment. Each experiment was independently repeated at least three times.

### 2.12. Apoptosis Assay

Cell apoptosis was assessed using Annexin V-FITC/PI staining according to the manufacturer’s protocol. After treatment, cells were harvested, washed, stained with Annexin V and PI, and analyzed by flow cytometry. Early and late apoptotic cells were quantified. Apoptosis-related proteins were further evaluated by Western blot.

### 2.13. Xenograft Experiments

All animal procedures were approved by the Institutional Animal Care and Use Committee of the Laboratory Animal Center of Zhengzhou University (approval no. ZZU-LAC20261324698; 27 March 2026). Male BALB/c nude mice (6–8 weeks old, 18–22 g) were obtained from GemPharmatech Co., Ltd. (Nanjing, China; production license No. SCXK (Su) 2018-0008) and housed under specific pathogen-free conditions in individually ventilated cages with a 12-h light/dark cycle at 22 ± 2 °C and 50 ± 10% humidity, with sterilized pelleted food and autoclaved drinking water provided ad libitum. After arrival, mice were acclimatized to the animal facility for 7 days before any experimental procedures were initiated. For the *DERL3* overexpression experiment, parental Caki-1 and 786-O cells stably overexpressing *DERL3* or vector control cells were harvested, resuspended in 100 μL of PBS, and subcutaneously injected at a density of 5 × 10^6^ cells per mouse into the flanks of nude mice (*n* = 5 per group, four groups total, 20 mice). Mice were randomly allocated to treatment groups using a computer-generated random number sequence (Excel, Microsoft Corp., Redmond, WA, USA). Randomization was performed when tumors reached approximately 80 mm^3^, and the allocation sequence was concealed from the investigators until group assignment. To minimize potential confounders, cages were randomly distributed across different racks in the animal facility, and treatments were administered in a staggered alternating sequence across groups to balance the time of administration. Tumor measurements were performed by an investigator blinded to treatment allocation, and all subsequent analyses were conducted on coded samples. For the therapeutic rescue experiment, resistant Caki-1-SR and 786-O-SR cells with *DERL3* knockdown or control were implanted into a separate cohort of nude mice (*n* = 5 per group, four groups total, 20 mice), followed by treatment with vehicle, sunitinib alone, Eeyarestatin I alone, or the combination of Eeyarestatin I and sunitinib. All animal experiments were performed once, with each xenograft tumor considered as one experimental unit, and the total number of mice used in this study was 40.

The a priori exclusion criteria were defined as follows: mice with tumor volume exceeding 2000 mm^3^, body weight loss greater than 20% of initial weight, or failure of tumor engraftment (tumor volume < 50 mm^3^ at day 7 post-inoculation) were to be excluded from the analysis. No mice met these exclusion criteria during the experiments; therefore, all tumor-bearing mice were included in the final analysis, and all data points from these animals were included in the statistical analysis. Tumor length and width were measured every 3 days using calipers, and tumor volume was calculated using the formula: volume = length × width^2^/2. At the experimental endpoint (day 28 of treatment or when tumors reached humane endpoints), mice were placed in a closed chamber and exposed to carbon dioxide (CO_2_) at a flow rate of 20% of the chamber volume per minute; after respiratory arrest was confirmed, cervical dislocation was performed as a secondary method to ensure death. This CO_2_ inhalation method is recommended by the American Veterinary Medical Association (AVMA) Guidelines for the Euthanasia of Animals (2020) for euthanasia of rodents, as it is rapid and minimizes distress, with cervical dislocation serving as an adjunctive confirmatory procedure. Following euthanasia, tumors were excised, weighed, and photographed.

For the in vivo experiments, group allocation was performed by an independent researcher who was not involved in subsequent animal handling or outcome assessment. The investigator responsible for drug administration and tumor measurement was blinded to treatment allocation throughout the entire experiment (from the start of treatment until the endpoint). Tumor volumes were measured and recorded using coded cage labels, and the blinding code was not broken until all measurements were completed. Data analysis was performed on fully coded datasets, with group identities revealed only after the final statistical analysis was concluded.

### 2.14. Outcome Measures

The primary experimental outcome measures were in vitro sunitinib IC50 (CCK-8 assay) and in vivo tumor volume and weight; these outcomes were used to justify the animal group size. Secondary experimental outcomes included apoptosis (Annexin V/PI), ROS levels (DCFH-DA), protein expression (*DERL3*, *DDIT3*, *CASP3*, *CASP6*, and *BAX*), and *DERL3* mRNA expression. Clinical outcomes were RECIST 1.1 response, progression-free survival (PFS), and overall survival (OS). All outcomes were specified before the final statistical analysis.

### 2.15. Statistical Analysis

No formal prospective sample-size calculation was performed for the retrospective clinical cohort or the in vitro experiments. Animal group sizes (*n* = 5 per group) were selected a priori based on prior experience and published ccRCC xenograft studies. Under an assumed 30% between-group difference in tumor volume and a 15% standard deviation, this group size was expected to provide approximately 80% power at a two-sided α of 0.05. For in vitro assays, at least three independent biological experiments were performed, each with three technical replicates; technical replicates were averaged so that the independent experiment, rather than the individual well, was the unit of statistical analysis.

Statistical analyses were performed using SPSS version 26.0, GraphPad Prism version 9.0, and R version 4.2.0. Continuous variables were assessed using Shapiro–Wilk tests and Q-Q plots, and homogeneity of variance was evaluated with Levene’s test. Normally distributed data are presented as mean ± standard deviation; non-normally distributed data are presented as median with interquartile range. Two-group comparisons used a two-sided unpaired Student’s *t* test (or Welch’s *t* test when variances were unequal); nonparametric data used the Mann–Whitney U test. Multiple-group comparisons used one-way ANOVA followed by Tukey’s post hoc test; Welch ANOVA with Games–Howell post hoc testing or Kruskal–Wallis testing with Dunn’s correction was used when assumptions were not met. Tumor growth curves were analyzed using two-way repeated-measures ANOVA.

Categorical variables were summarized as counts and percentages and compared using the chi-square test or Fisher’s exact test, as appropriate. Kaplan–Meier curves for PFS and OS were compared using the log-rank test, and numbers at risk were displayed at prespecified time points. Univariate and multivariate Cox proportional hazards models were used to estimate hazard ratios and 95% confidence intervals. Variables with *p* < 0.05 in univariate analysis and clinically relevant variables were considered for multivariable modeling. The proportional-hazards assumption was assessed using Schoenfeld residuals and log-minus-log survival plots. Analyses used complete available cases without imputation. All tests were two-sided, and *p* < 0.05 was considered statistically significant.

### 2.16. Reproducibility, Reporting, and Nomenclature

The exact biological replicate number is stated in each figure legend; unless otherwise specified, *n* denotes independent biological experiments or independent animals rather than technical replicates. Technical replicates were averaged before inferential testing. No data point was excluded unless it met a prespecified exclusion criterion. Statistical tests, post hoc procedures, and significance symbols are defined in the Methods and figure legends. The terms mRNA, gene, or protein are used where needed to avoid ambiguity.

## 3. Results

### 3.1. Serial In Vivo Sunitinib Selection Generates Stable Resistant ccRCC Cell Models

**Figure 1:**
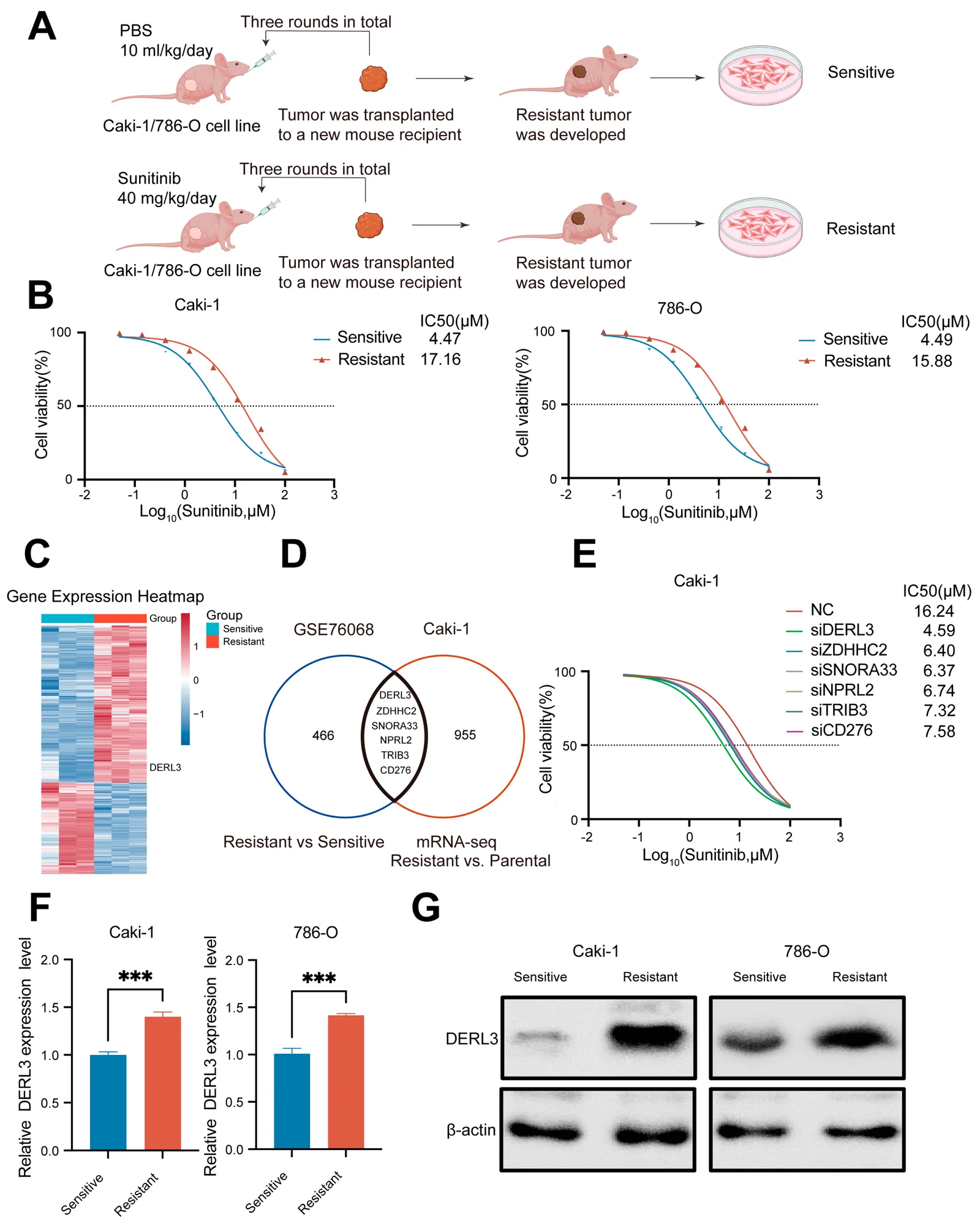
Serial in vivo selection and integrated transcriptomic screening identify *DERL3* as a key mediator of acquired sunitinib resistance in ccRCC. (**A**) Schematic illustration of the establishment of sunitinib-sensitive and sunitinib-resistant ccRCC cell models. Caki-1 and 786-O cells were independently implanted subcutaneously into nude mice and assigned to two parallel groups. Xenografts in the sensitive group received PBS (10 mL/kg/day), whereas those in the resistant group received sunitinib (40 mg/kg/day). Tumors from each group were harvested and serially transplanted into new recipient mice for three consecutive rounds. The resulting sensitive and resistant groups each contained both Caki-1- and 786-O-derived cells. (**B**) IC50 analysis of parental and sunitinib-resistant Caki-1 and 786-O cells (*n* = 3 independent experiments). Data are mean ± SD; two-sided unpaired Student’s *t* tests compared each resistant derivative with its matched parental control. (**C**) Transcriptomic profiling of parental and resistant ccRCC cells (*n* = 3 independent biological replicates per group). The heatmap shows genes meeting adjusted *p* < 0.05 and |log2 fold change| > 1. (**D**) Integrated analysis of RNA-seq data from resistant ccRCC cells and GSE76068. Six concordantly upregulated genes (*DERL3*, *ZDHHC2*, *SNORA33*, *NPRL2*, *TRIB3*, and *CD276*) were identified. (**E**) siRNA-based functional screening of the six candidates (*n* = 3 independent experiments per gene). IC50 values were compared by one-way ANOVA with Tukey’s post hoc test. (**F**,**G**) qRT-PCR (**F**) and Western blot (**G**) validation of *DERL3* mRNA and protein expression in parental and resistant cells (*n* = 3 independent experiments). Data are mean ± SD; two-sided unpaired Student’s *t* tests were used. * *p* < 0.05; ** *p* < 0.01; *** *p* < 0.001.

To model acquired sunitinib resistance under physiologically relevant drug pressure, we established an in vivo serial selection system using Caki-1 and 786-O xenografts. Parental cells were implanted into nude mice, and sunitinib was administered by oral gavage at 40 mg/kg/day after tumor establishment. Residual tumors surviving sunitinib treatment were harvested, dissociated into viable tumor cells, expanded, and re-implanted into new mice. After three rounds of drug selection, stable resistant derivatives were obtained and designated Caki-1-SR and 786-O-SR (Figure 1A). Compared with parental cells, Caki-1-SR and 786-O-SR cells exhibited markedly reduced sensitivity to sunitinib. IC50 assays showed that the IC50 values of resistant cells were significantly higher than those of their corresponding parental controls (Figure 1B). These results confirmed the successful establishment of stable sunitinib-resistant ccRCC cell models. To identify molecular alterations associated with acquired resistance, we performed RNA sequencing of parental and resistant ccRCC cells, with three biological replicates per group. Differential expression analysis identified a set of genes significantly upregulated or downregulated in resistant cells (Figure 1C). To prioritize clinically relevant candidates, we intersected the upregulated genes from our resistant cell models with the sunitinib-resistant ccRCC patient dataset GSE76068. Six genes, including *DERL3*, *ZDHHC2*, *SNORA33*, *NPRL2*, *TRIB3* and *CD276* were consistently upregulated in resistant models and the external dataset (Figure 1D). These genes were selected for functional screening. Using siRNA-mediated knockdown, we assessed the contribution of each candidate gene to sunitinib resistance. Among the six genes tested, *DERL3* knockdown produced the most pronounced decrease in sunitinib IC50, indicating that *DERL3* may be a functional mediator rather than a passive marker of resistance (Figure 1E and Supplementary Figure S1A). qRT-PCR and Western blot further confirmed that *DERL3* mRNA and protein levels were significantly elevated in Caki-1-SR and 786-O-SR cells compared with parental cells (Figure 1F,G). Together, these findings identified *DERL3* as a candidate driver of acquired sunitinib resistance in ccRCC.

### 3.2. DERL3 Is Upregulated in Sunitinib-Resistant ccRCC Specimens and Is Associated with Aggressive Clinicopathological Features and Poor Prognosis

**Figure 2:**
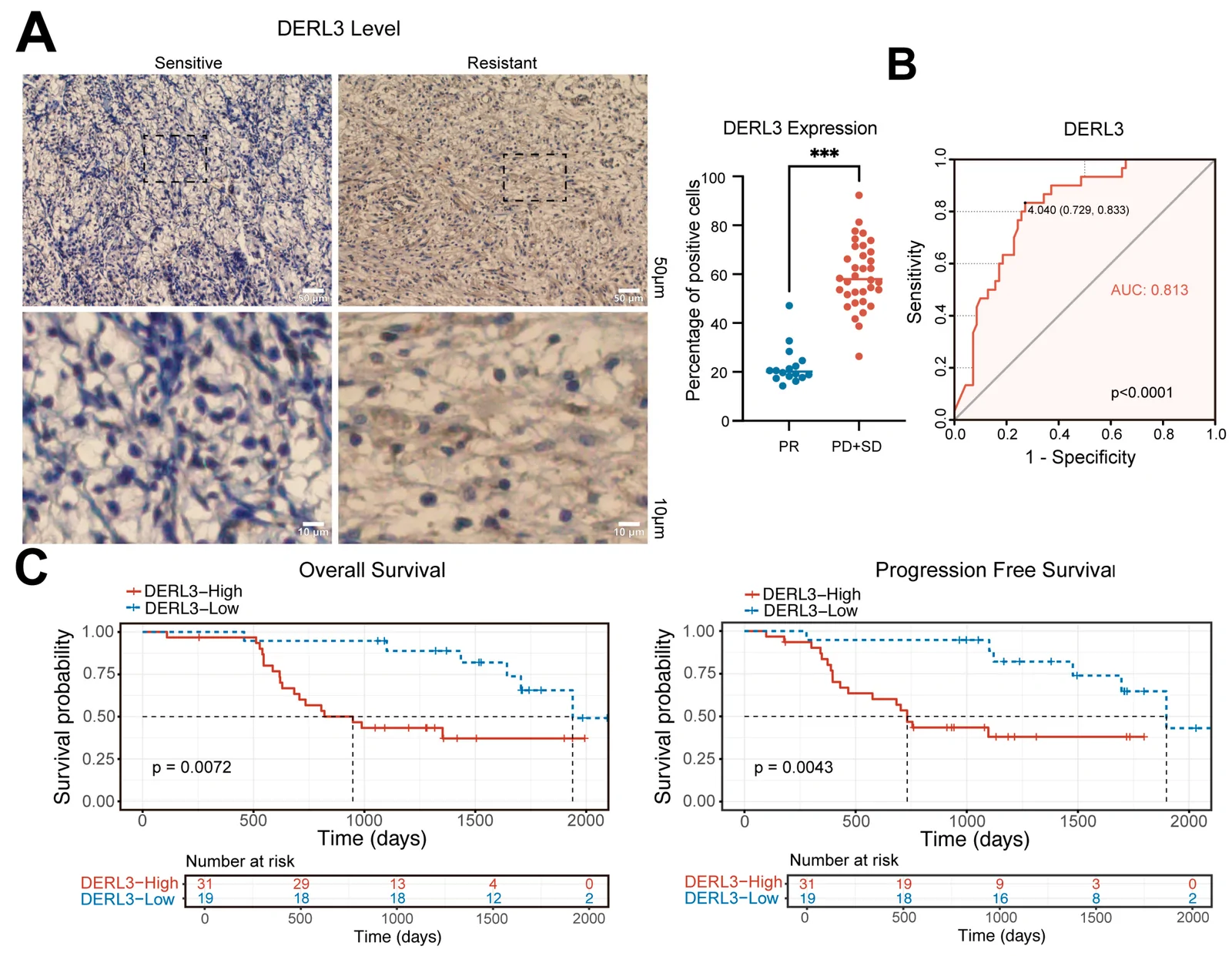
*DERL3* is upregulated in sunitinib-resistant ccRCC specimens and predicts poor clinical outcome. (**A**) Representative *DERL3* immunohistochemistry in tumors with PR versus SD or PD, with blinded quantification of the percentage of positively stained tumor cells. Individual-patient values are shown with the median. (**B**) ROC analysis of *DERL3* expression for unfavorable response (PD or SD versus PR; *n* = 50). AUC = 0.813; threshold = 4.040; specificity = 0.729; sensitivity = 0.833; *p* < 0.0001. (**C**) Kaplan–Meier PFS and OS curves for *DERL3*-low (*n* = 19) and *DERL3*-high (*n* = 31) groups. Two-sided log-rank tests were used; censoring marks and numbers at risk at 0, 500, 1000, 1500, and 2000 days are shown. * *p* < 0.05; ** *p* < 0.01; *** *p* < 0.001.

We next evaluated the clinical relevance of *DERL3* in ccRCC specimens from patients treated with neoadjuvant sunitinib. Immunohistochemical staining demonstrated higher *DERL3* protein expression in tumors with poor response or resistance to sunitinib than in sensitive tumors (Figure 2A). *DERL3* staining was predominantly cytoplasmic, consistent with its endoplasmic reticulum-associated localization. ROC analysis of the prespecified unfavorable-response endpoint (PD or SD versus PR) yielded an AUC of 0.813. The classification threshold used in the analysis was 4.040, with specificity 0.729 and sensitivity 0.833 (*p* < 0.0001) (Figure 2B). Based on the study’s classification procedure, 19 patients were assigned to the *DERL3*-low group and 31 to the *DERL3*-high group. *DERL3* expression was not associated with gender, age, or tumor laterality, but high expression was associated with larger tumor size, higher WHO/ISUP grade, tumor necrosis, advanced T and AJCC stage, and poorer response (Table 1). Kaplan–Meier analysis showed shorter PFS and OS in the *DERL3*-high group, with numbers at risk displayed below each curve (Figure 2C). In multivariable Cox models, *DERL3*-high expression remained associated with PFS (*HR* = 2.68, 95% *CI*: 1.12–6.41, *p* = 0.027) and OS (*HR* = 2.91, 95% *CI*: 1.08–7.82, *p* = 0.035) (Table 2). Public-dataset analyses showed higher *DERL3* expression in ccRCC and an association with inferior survival (Supplementary Figure S2). These preliminary findings support *DERL3* as a candidate response-associated and prognostic biomarker for future validation.

### 3.3. DERL3 Overexpression Reduces Sunitinib Sensitivity, Whereas DERL3 Knockdown Restores Sensitivity in Resistant ccRCC Cells

**Figure 3:**
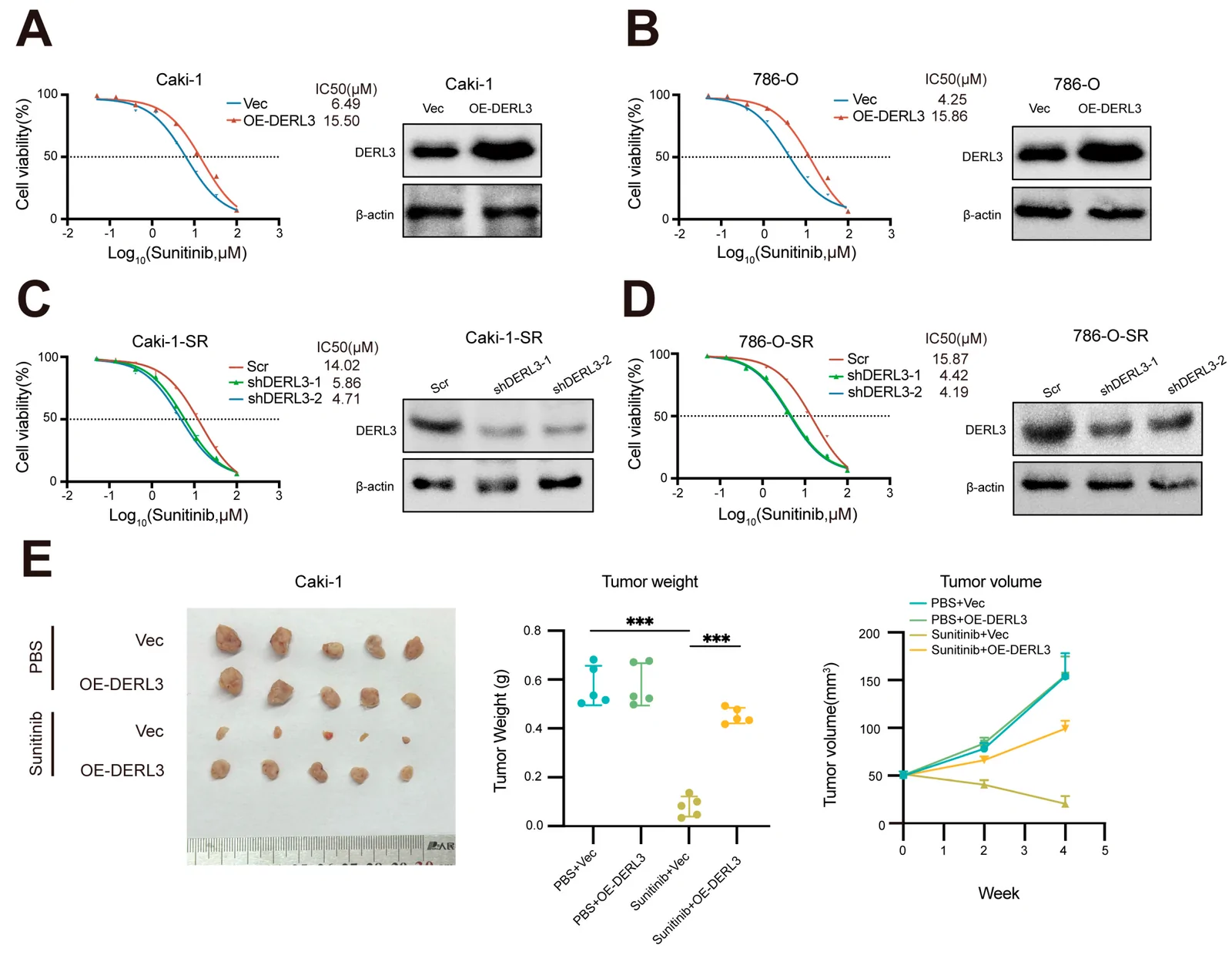
*DERL3* overexpression reduces sunitinib sensitivity, whereas *DERL3* knockdown restores sensitivity in resistant ccRCC cells. (**A**,**B**) *DERL3* was overexpressed in parental sunitinib-sensitive Caki-1 (*n* = 3 independent experiments) (**A**) and 786-O (*n* = 3 independent experiments) (**B**) cells. Western blot confirmed *DERL3* overexpression, and IC50 analysis was performed to evaluate the effect of *DERL3* overexpression on sunitinib sensitivity. *DERL3* overexpression increased the IC50 of sunitinib in both cell lines. (**C**,**D**) *DERL3* was knocked down using two independent shRNAs in sunitinib-resistant Caki-1-SR (*n* = 3 independent experiments) (**C**) and 786-O-SR (*n* = 3 independent experiments) (**D**) cells. Western blot confirmed the knockdown efficiency of sh*DERL3*-1 and sh*DERL3*-2, and IC50 analysis showed that *DERL3* knockdown restored sunitinib sensitivity in resistant ccRCC cells. Data are mean ± SD; two-group comparisons used two-sided unpaired Student’s *t* tests and comparisons involving multiple constructs used one-way ANOVA with Tukey’s post hoc test. (**E**) Xenograft evaluation of *DERL3* overexpression under vehicle or sunitinib treatment (*n* = 5 mice per group). Tumor growth was analyzed by two-way repeated-measures ANOVA with Šídák-adjusted comparisons; endpoint tumor weights were analyzed by one-way ANOVA with Tukey’s post hoc test. Data are mean ± SD. * *p* < 0.05; ** *p* < 0.01; *** *p* < 0.001.

To determine whether *DERL3* is sufficient to promote sunitinib resistance, we overexpressed *DERL3* in parental Caki-1 and 786-O cells. Western blot confirmed robust *DERL3* overexpression (Figure 3A,B). Functional assays showed that *DERL3* overexpression significantly increased the IC50 of sunitinib in both cell lines, indicating reduced drug sensitivity (Figure 3A,B). Conversely, to determine whether *DERL3* is required for maintenance of the resistant phenotype, we silenced *DERL3* in Caki-1-SR and 786-O-SR cells using two independent shRNAs. Western blotting confirmed efficient *DERL3* knockdown (Figure 3C,D). *DERL3* depletion markedly reduced the IC50 of sunitinib in resistant cells, demonstrating restoration of drug sensitivity (Figure 3C,D). The consistent effects of two independent shRNAs reduced the likelihood of off-target effects. We further evaluated the role of *DERL3* in vivo. Parental ccRCC cells overexpressing *DERL3* or vector control were implanted into nude mice and treated with vehicle or sunitinib after tumor establishment. In vector-control tumors, sunitinib significantly suppressed tumor growth. In contrast, *DERL3*-overexpressing tumors exhibited attenuated response to sunitinib, as reflected by larger tumor volumes and heavier tumor weights under sunitinib treatment compared with vector-control tumors (Figure 3E). These results demonstrate that *DERL3* overexpression is sufficient to reduce sunitinib sensitivity in vivo.

### 3.4. DERL3 Knockdown Activates Pro-Apoptotic ER Stress and ROS-Dependent Apoptosis in Resistant ccRCC Cells

**Figure 4:**
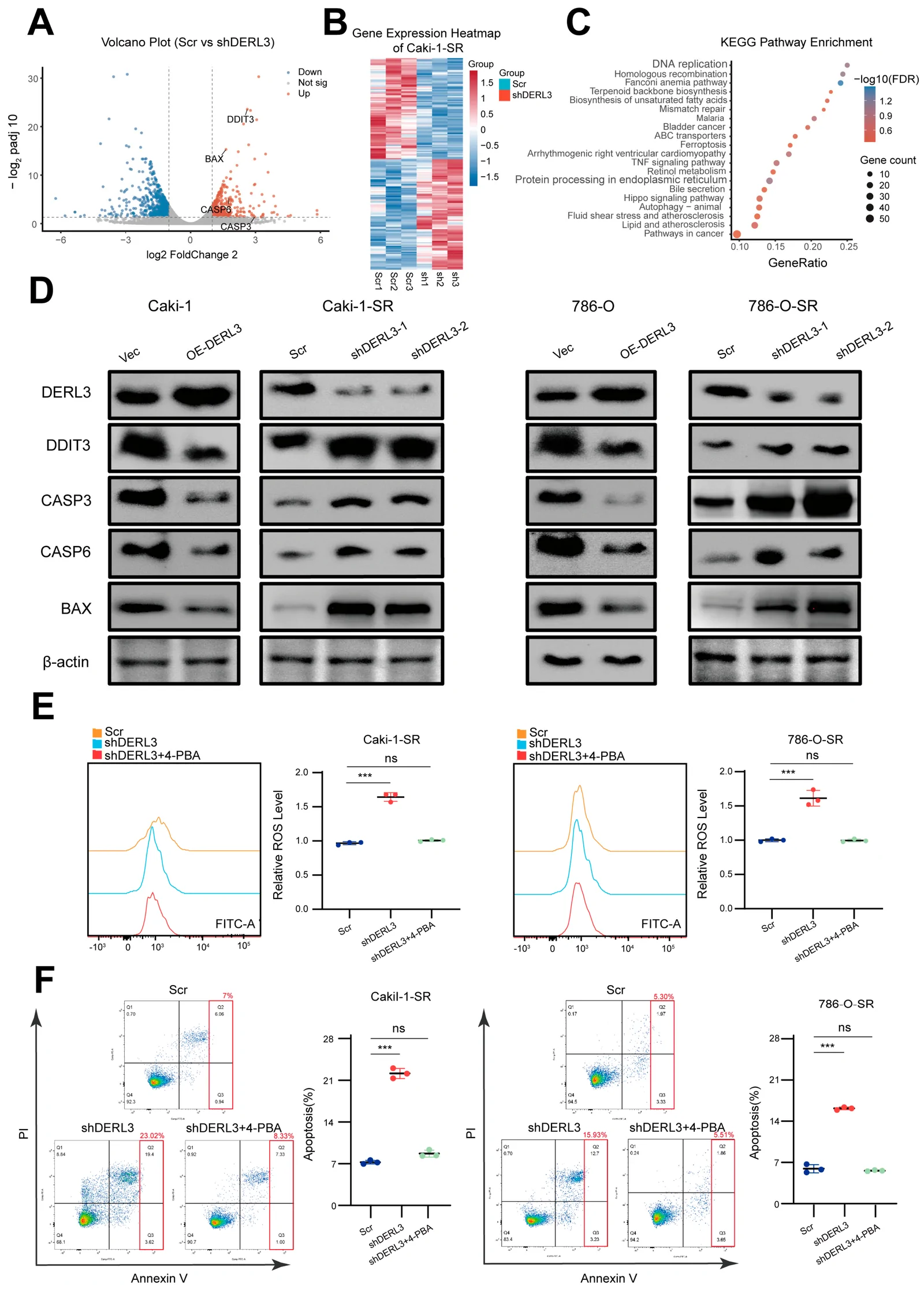
*DERL3* promotes sunitinib resistance by suppressing pro-apoptotic ER stress, ROS accumulation, and apoptosis. (**A**) Volcano plot showing differentially expressed genes in resistant ccRCC cells after *DERL3* knockdown. (**B**) Heatmap showing representative genes altered by *DERL3* depletion in resistant ccRCC cells. (**C**) Functional enrichment analysis showing activation of endoplasmic reticulum stress- and DNA damage-related pathways after *DERL3* knockdown. (**D**) Western blot analysis of ER stress- and apoptosis-related proteins. *DERL3* overexpression suppressed pro-apoptotic ER stress and apoptosis-related markers, whereas *DERL3* knockdown increased *DDIT3*, *CASP3*, *CASP6*, and *BAX* expression in resistant cells treated with sunitinib. (**E**,**F**) ROS detection and apoptosis analysis after *DERL3* knockdown. *DERL3* depletion increased intracellular ROS accumulation and Annexin V-positive apoptosis, whereas inhibition of ER stress reduced ROS generation and partially rescued apoptosis in *DERL3*-silenced resistant cells. Data are shown as *n* = 3 per group. Data are mean ± SD; multi-group comparisons used one-way ANOVA with Tukey’s post hoc test. ns, not significant (*p* ≥ 0.05); * *p* < 0.05; ** *p* < 0.01; *** *p* < 0.001.

To explore the downstream mechanisms by which *DERL3* regulates sunitinib resistance, we performed RNA sequencing in resistant ccRCC cells after *DERL3* knockdown. Volcano plot and heatmap analyses showed broad transcriptomic alterations following *DERL3* silencing (Figure 4A,B). Functional enrichment analysis revealed significant activation of pathways related to endoplasmic reticulum stress and DNA damage response (Figure 4C). Given that *DERL3* is an ERAD-related protein, we focused on the ER stress response. Western blot showed that *DERL3* overexpression suppressed markers of pro-apoptotic ER stress and apoptosis after sunitinib exposure, including *DDIT3*, *CASP3*, *CASP6*, and *BAX*. In contrast, *DERL3* knockdown increased *DDIT3* expression and enhanced apoptosis-related protein activation in resistant cells treated with sunitinib (Figure 4D). These findings suggest that *DERL3* promotes resistance by limiting pro-apoptotic ER stress signaling and downstream apoptotic execution. Because unresolved ER stress can induce oxidative injury and apoptosis, we next examined whether *DERL3* regulates ROS accumulation in resistant ccRCC cells. DCFH-DA staining showed that *DERL3* knockdown significantly increased intracellular ROS levels, particularly after sunitinib treatment (Figure 4E). This increase was accompanied by enhanced Annexin V-positive apoptosis (Figure 4F). To determine whether ROS accumulation and apoptosis were dependent on ER stress activation, *DERL3*-silenced cells were treated with an ER stress inhibitor (Figure 4E,F). ER stress inhibition reduced ROS accumulation in *DERL3*-knockdown cells and partially rescued cells from apoptosis (Figure 4E,F). These data indicate that *DERL3* maintains sunitinib resistance, at least in part, by suppressing an ER stress-ROS-apoptosis axis.

### 3.5. DERL3 Expression Predicts Poor Sunitinib Response and DERL3-Targeted Inhibition Restores Drug Sensitivity in Resistant ccRCC Xenografts

**Figure 5:**
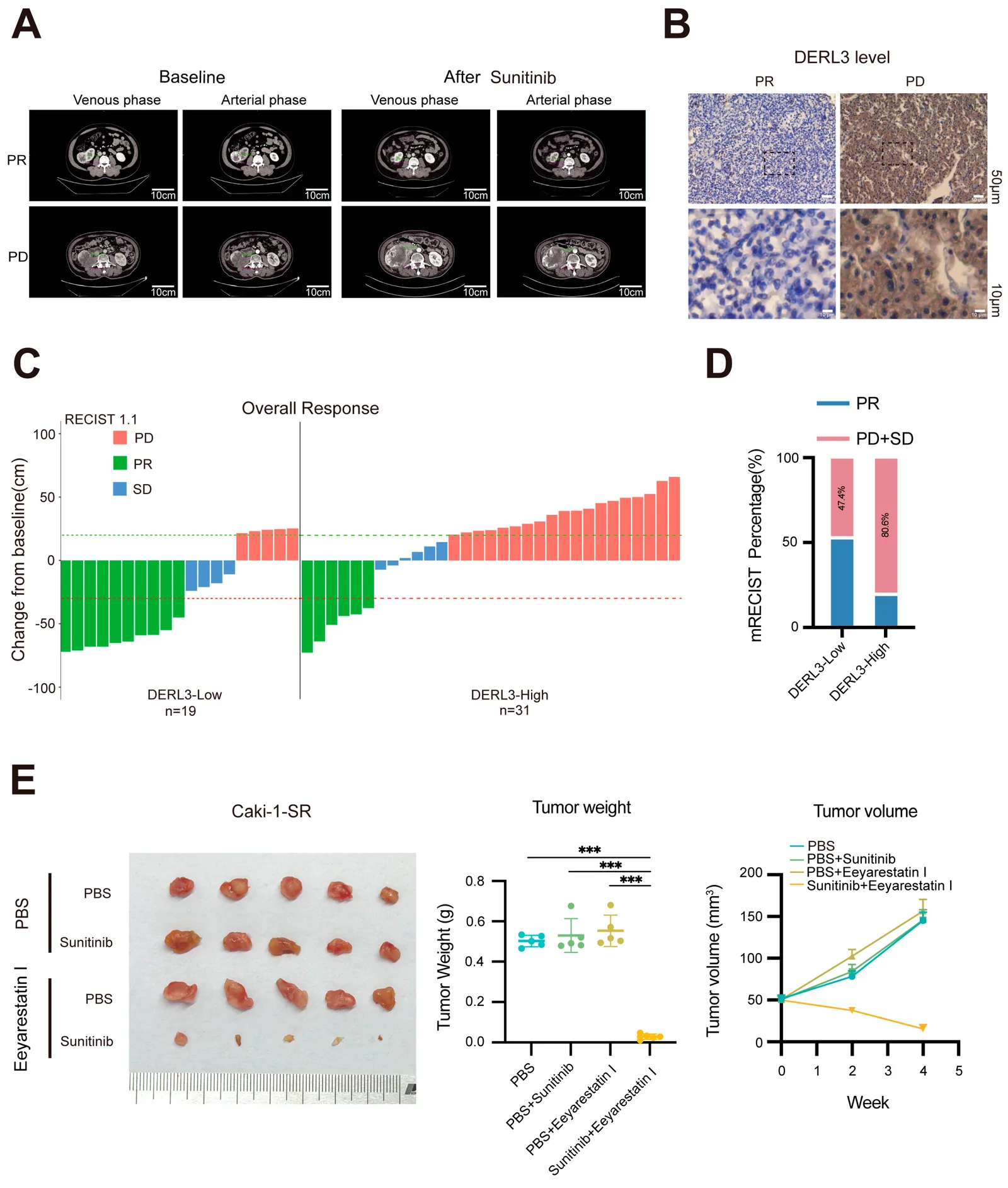
*DERL3* expression is associated with poor response to neoadjuvant sunitinib and an ERAD-targeting vulnerability in ccRCC. (**A**) Representative contrast-enhanced CT images showing tumor changes before and after neoadjuvant sunitinib treatment. Arterial-phase and venous-phase images were used to evaluate radiographic tumor response and tumor shrinkage after treatment. (**B**) Representative immunohistochemical staining and quantification of *DERL3* expression in tumor specimens from patients with partial response (PR) or progressive disease (PD) after neoadjuvant sunitinib treatment. *DERL3* expression was higher in PD tumors than in PR tumors; two-sided unpaired Student’s *t* test. (**C**) Waterfall plot showing the percentage change in tumor size after neoadjuvant sunitinib treatment according to *DERL3* expression status. *DERL3*-high tumors exhibited poorer tumor shrinkage than *DERL3*-low tumors. (**D**) Stacked bar plot showing the distribution of treatment responses in *DERL3*-low (*n* = 19) and *DERL3*-high (*n* = 31) groups. Patients with high *DERL3* expression showed a higher proportion of unfavorable responses; chi-square or Fisher’s exact test, as appropriate. (**E**) Resistant Caki-1-SR xenografts treated with vehicle, sunitinib, Eeyarestatin I, or the combination (*n* = 5 mice per group). Tumor growth used two-way repeated-measures ANOVA with Šídák-adjusted comparisons; endpoint weights used one-way ANOVA with Tukey’s post hoc test. Data are mean ± SD. * *p* < 0.05; ** *p* < 0.01; *** *p* < 0.001.

To further define the clinical and therapeutic relevance of *DERL3* in sunitinib-resistant ccRCC, we first evaluated *DERL3* expression in tumor specimens from patients who received neoadjuvant sunitinib. Immunohistochemical analysis showed that *DERL3* expression was markedly higher in resistant tumors than in sensitive tumors (Figure 5A,B). Waterfall and response-distribution analyses demonstrated poorer tumor shrinkage in *DERL3*-high tumors (Figure 5C,D). We next tested whether perturbing ERAD could increase sunitinib sensitivity in vivo. Resistant Caki-1-SR xenografts were treated with vehicle, sunitinib, the ERAD inhibitor Eeyarestatin I, or the combination. Sunitinib monotherapy produced limited suppression and Eeyarestatin I alone had little effect, whereas the combination produced the largest reduction in tumor volume and weight (Figure 5E). Collectively, these results indicate that *DERL3* not only predicts poor clinical response to sunitinib but also represents a potential therapeutic target for overcoming acquired sunitinib resistance in ccRCC.

## 4. Discussion

In this study, we identified *DERL3* as a functional mediator of acquired sunitinib resistance in ccRCC. By combining serial in vivo selection, RNA sequencing, external dataset integration, functional screening, clinical specimen validation, and mechanistic experiments, we provide evidence that *DERL3* is not merely associated with resistance but actively contributes to the resistant phenotype. *DERL3* was consistently upregulated in resistant ccRCC models, in a sunitinib-resistant ccRCC patient-derived xenograft dataset, and in resistant clinical specimens. High *DERL3* expression predicted poor response to neoadjuvant sunitinib and inferior survival outcomes. Functionally, *DERL3* overexpression reduced sunitinib sensitivity, whereas *DERL3* silencing restored sensitivity in resistant cells and xenografts. Mechanistically, *DERL3* appeared to suppress pro-apoptotic ER stress, ROS accumulation, and caspase-dependent apoptosis, thereby enabling ccRCC cells to survive under sunitinib pressure.

Sunitinib resistance remains clinically and biologically relevant in ccRCC. Although ICI-based combinations have reshaped first-line treatment, VEGFR-targeted TKIs remain integral components of ICI-TKI regimens and remain options when immunotherapy is unsuitable or unavailable [9,13,14,39,40]. Recent studies continue to identify diverse sunitinib-adaptive programs, including ferroptosis suppression, GCN2-ATF4-dependent serine synthesis, and ubiquitin-dependent stabilization of HIF2α [22,23,24]. These findings support the continued use of sunitinib as a model for VEGFR-TKI adaptation and provide a basis for examining whether *DERL3*-associated stress adaptation also influences response to contemporary combination treatment.

A major strength of our study is the use of serial in vivo drug selection to generate resistant ccRCC cells. Many resistance models are established by chronic in vitro exposure to increasing drug concentrations, which may not fully capture the influence of tumor microenvironment, vascular stress, nutrient limitation, and in vivo pharmacologic pressure [20,41,42,43]. Our approach used repeated xenograft selection under sunitinib treatment, followed by tumor dissociation and re-implantation, thereby enriching tumor cells capable of surviving drug pressure in vivo. Integration with GSE76068 further strengthened candidate prioritization by focusing on genes shared between our experimental models and a clinically derived ccRCC xenograft resistance dataset.

*DERL3* is an ER membrane protein involved in ERAD, a protein quality-control pathway that removes misfolded glycoproteins from the ER for proteasomal degradation [32,33]. Under stress conditions, ERAD can be cytoprotective by reducing proteotoxic burden and preventing lethal ER stress. Our findings suggest that ccRCC cells may exploit *DERL3* upregulation to buffer sunitinib-induced stress and avoid apoptosis. Importantly, pharmacologic inhibition of ER stress partially reduced ROS and apoptosis induced by *DERL3* knockdown, supporting the mechanistic link between *DERL3*, ER stress, oxidative injury, and cell death [25,29,33,36]. Importantly, pharmacologic inhibition of ER stress partially reduced ROS and apoptosis induced by *DERL3* knockdown, supporting the mechanistic link between *DERL3*, ER stress, oxidative injury, and cell death.

The relationship between ER stress and cancer therapy response is context dependent. Mild or adaptive UPR activation can promote tumor survival, whereas severe or unresolved ER stress can trigger apoptosis [28,35]. This dual role may explain why *DERL3*-mediated ERAD is advantageous for resistant ccRCC cells [32,33]. By facilitating ER protein quality control, *DERL3* may reduce the intensity of pro-apoptotic ER stress and prevent ROS-dependent cell death during sunitinib treatment [32,36]. Therefore, *DERL3* inhibition may lower the threshold for sunitinib-induced apoptosis and restore drug sensitivity.

Our clinical observations represent a preliminary exploration of intratumoral *DERL3* as a response-associated biomarker for sunitinib. In contemporary ICI-TKI therapy, a plausible mechanistic framework is that VEGFR inhibition may normalize abnormal tumor vasculature, reduce hypoxia, and facilitate immune cell infiltration, whereas immune checkpoint blockade restores antitumor T-cell activity; these processes may interact with tumor-cell ER stress, proteostasis, ROS homeostasis, and the immune microenvironment. *DERL3* could therefore influence combination efficacy by altering stress tolerance during TKI exposure and, indirectly, immune susceptibility. Future validation will use prespecified assays and cutoffs in large prospective multicenter cohorts. We plan prospective clinical trials that stratify patients by pretreatment *DERL3* status and include relevant TKI-monotherapy and ICI-TKI cohorts to test the biomarker-treatment interaction.

This study provides a preliminary clinical and mechanistic framework for *DERL3*-mediated sunitinib resistance. The next phase will include large prospective multicenter cohorts, orthotopic and immune-competent models, development of selective *DERL3*-directed approaches, and deeper investigation of the upstream regulation and downstream ERAD substrates of *DERL3*. These studies will establish the generalizability and mechanistic depth of the present findings and support planned clinical evaluation in contemporary treatment settings.

Several limitations should nevertheless be acknowledged. First, the clinical cohort included only 50 patients from a single center and was retrospective, creating potential risks of selection bias, imprecise effect estimates, and overfitting. Second, the neoadjuvant sunitinib population does not represent the full spectrum of current advanced-ccRCC treatment, and the findings require further validation in patients receiving contemporary ICI-TKI combinations. Third, subcutaneous xenografts in immunodeficient mice do not fully reproduce the orthotopic renal microenvironment or the immune contribution to therapeutic response. Consequently, our future work will incorporate genetic rescue experiments, selective *DERL3*-directed agents, orthotopic and immune-competent models, and prospective multicenter cohorts.

In conclusion, our study identifies *DERL3* as a clinically relevant driver of acquired sunitinib resistance in ccRCC. *DERL3* promotes resistance by suppressing pro-apoptotic ER stress, ROS accumulation, and apoptosis. Targeting *DERL3* restores sunitinib sensitivity in resistant models, providing a potential therapeutic strategy for overcoming VEGFR-TKI resistance in ccRCC.

## 5. Conclusions

In summary, this study identifies *DERL3* as a clinically relevant functional driver of acquired sunitinib resistance in clear cell renal cell carcinoma. By combining serial in vivo selection, transcriptomic integration, functional screening, and mechanistic dissection, we demonstrate that *DERL3* is consistently upregulated in resistant models and patient specimens, correlates with poor treatment response and shorter survival, and actively promotes resistance through suppression of pro-apoptotic endoplasmic reticulum stress, ROS accumulation, and caspase dependent apoptosis. Conversely, *DERL3* knockdown or pharmacological inhibition unleashes ER stress, elevates ROS levels, and restores sunitinib sensitivity both in vitro and in vivo. These findings position *DERL3* as a promising predictive biomarker for VEGFR-TKI responsiveness and a potential therapeutic target for overcoming acquired resistance. Targeting the *DERL3*–ER stress–ROS axis may offer a novel strategy to enhance the efficacy of antiangiogenic therapy in ccRCC. These findings provide a rationale for future large multicenter validation and prospective clinical trials in contemporary TKI and ICI-TKI settings, together with deeper investigation of *DERL3* upstream regulation and downstream ERAD substrates.

## Figures and Tables

**Table 1:** Association between *DERL3* expression and clinicopathological characteristics in patients with ccRCC treated with neoadjuvant sunitinib.

Variables	*DERL3*-Low (*n* = 19)	*DERL3*-High (*n* = 31)	*p*-Value
**Gender**			0.945
Female	9 (47.4%)	15 (48.4%)	
Male	10 (52.6%)	16 (51.6%)	
**Age (years)**			0.659
<40	3 (15.8%)	3 (9.7%)	
≥40	16 (84.2%)	28 (90.3%)	
**Laterality**			0.946
Left	9 (47.4%)	15 (48.4%)	
Right	10 (52.6%)	16 (51.6%)	
**Tumor size**			0.043
<7 cm	12 (63.2%)	10 (32.3%)	
≥7 cm	7 (36.8%)	21 (67.7%)	
**Tumor number**			0.452
Single	17 (89.5%)	24 (77.4%)	
Multiple	2 (10.5%)	7 (22.6%)	
**WHO/ISUP grade**			0.020
Grade 1–2	13 (68.4%)	10 (32.3%)	
Grade 3–4	6 (31.6%)	21 (67.7%)	
**Tumor necrosis**			0.042
Absent	14 (73.7%)	13 (41.9%)	
Present	5 (26.3%)	18 (58.1%)	
**Sarcomatoid/rhabdoid differentiation**			0.067
Absent	18 (94.7%)	22 (71.0%)	
Present	1 (5.3%)	9 (29.0%)	
**Lymphovascular invasion**			0.074
Absent	15 (78.9%)	16 (51.6%)	
Present	4 (21.1%)	15 (48.4%)	
**Venous tumor thrombus**			0.063
Absent	16 (84.2%)	17 (54.8%)	
Present	3 (15.8%)	14 (45.2%)	
**T stage**			0.042
T1	8 (42.1%)	6 (19.4%)	
T2	6 (31.6%)	11 (35.5%)	
T3	4 (21.1%)	9 (29.0%)	
T4	1 (5.3%)	5 (16.1%)	
**N stage**			0.221
N0	15 (78.9%)	18 (58.1%)	
N1	4 (21.1%)	13 (41.9%)	
**AJCC stage**			0.036
Stage I	8 (42.1%)	5 (16.1%)	
Stage II	5 (26.3%)	7 (22.6%)	
Stage III	5 (26.3%)	10 (32.3%)	
Stage IV	1 (5.3%)	9 (29.0%)	
**Best response to neoadjuvant sunitinib**			0.032
PR	10 (52.6%)	6 (19.4%)	
SD	4 (21.1%)	6 (19.4%)	
PD	5 (26.3%)	19 (61.2%)	

Abbreviations: ccRCC, renal cell carcinoma; WHO/ISUP, World Health Organization/International Society of Urological Pathology; AJCC, American Joint Committee on Cancer; PR, partial response; SD, stable disease; PD, progressive disease. Figure categorical response analysis: best response was classified as PR, SD, or PD according to RECIST 1.1. Categorical variables were compared using the chi-square test or Fisher’s exact test, as appropriate.

**Table 2:** Cox regression analyses of prognostic factors for progression-free survival and overall survival in patients with ccRCC treated with neoadjuvant sunitinib.

Variables	PFS Univariate HR (95% CI)	*p*-Value	PFS Multivariate HR (95% CI)	*p*-Value	OS Univariate HR (95% CI)	*p*-Value	OS Multivariate HR (95% CI)	*p*-Value
Age ≥40 vs. <40 years	1.21 (0.48–3.08)	0.684			1.34 (0.47–3.80)	0.586		
Male vs. Female	1.08 (0.51–2.29)	0.842			1.15 (0.50–2.64)	0.742		
Tumor size ≥7 cm vs. <7 cm	1.86 (0.91–3.82)	0.089			2.05 (0.92–4.58)	0.079		
WHO/ISUP grade 3–4 vs. 1–2	2.12 (1.02–4.42)	0.044	1.61 (0.72–3.60)	0.246	2.36 (1.04–5.37)	0.041	1.76 (0.72–4.31)	0.217
Tumor necrosis present vs. absent	1.94 (0.94–4.02)	0.074			2.21(0.98–5.00)	0.056		
AJCC stage III–IV vs. I–II	2.43 (1.16–5.10)	0.019	1.74 (0.76–3.99)	0.191	2.89 (1.25–6.69)	0.013	1.96 (0.76–5.06)	0.163
*DERL3* high vs. low	3.26 (1.47–7.24)	0.004	2.68 (1.12–6.41)	0.027	3.74 (1.51–9.27)	0.004	2.91 (1.08–7.82)	0.035

## Data Availability

The datasets generated during the current study are available from the corresponding author upon reasonable request. The dataset supporting the conclusions of this article is available in the Gene Expression Omnibus repository under accession number GSE76068 (https://www.ncbi.nlm.nih.gov/geo/query/acc.cgi?acc=GSE76068, accessed on 5 August 2026).

## Supplementary Materials

The following supporting information can be downloaded at https://www.mdpi.com/article/10.3390/biomedicines14081841/s1, Figure S1: Functional screening and validation of candidate resistance genes; Figure S2: Public dataset analysis of *DERL3* expression and prognosis in ccRCC; Table S1: siRNA sequences used for candidate resistance gene screening; Table S2: Primer sequences used for qRT-PCR.

## Abbreviations

RCC	Renal Cell Carcinoma
ccRCC	clear cell Renal Cell Carcinoma
VEGF	Vascular Endothelial Growth Factor
VEGFR	Vascular Endothelial Growth Factor Receptor
PDGFR	Platelet-Derived Growth Factor Receptors
ICI	Immune Checkpoint Inhibitor
ERAD	Endoplasmic Reticulum-Associated Degradation
UPR	Unfolded Protein Response
CCK-8	Cell Counting Kit-8
DMSO	Dimethyl Sulfoxide
PR	Partial Response
PD	Progressive Disease
SD	Stable Disease
ROC	Receiver Operating Characteristic
4-PBA	4-Phenylbutyric Acid
ATCC	American Type Culture Collection
FBS	Fetal Bovine Serum
