# Supplementary material for: DERL3 Predicts and Drives Acquired Sunitinib Resistance in Renal Cell Carcinoma by Suppressing ER Stress–ROS-Dependent Apoptosis
Source: Biomedicines. 2026 Aug 15;14(8):1841. doi: 10.3390/biomedicines14081841 (PMC13509321; doi:10.3390/biomedicines14081841)
Supplement: Supplementary file 1 [file biomedicines-14-01841-s001.zip › biomedicines-4442914-supplementary.pdf]

Supplement

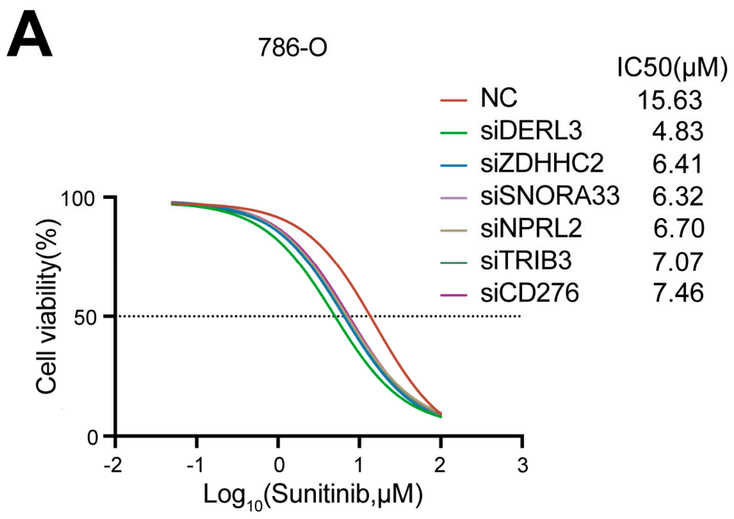

Figure S1. *Functional screening and validation of candidate resistance genes.* (**A**) siRNA-mediated knockdown of the six overlapping upregulated genes identified by integrated transcriptomic analysis. DERL3 knockdown most strongly reduced sunitinib IC50 (n = 3 independent experiments per gene). Data are mean ± SD; one-way ANOVA with Tukey's post hoc test. *P < 0.05; **P < 0.01; ***P < 0.001.

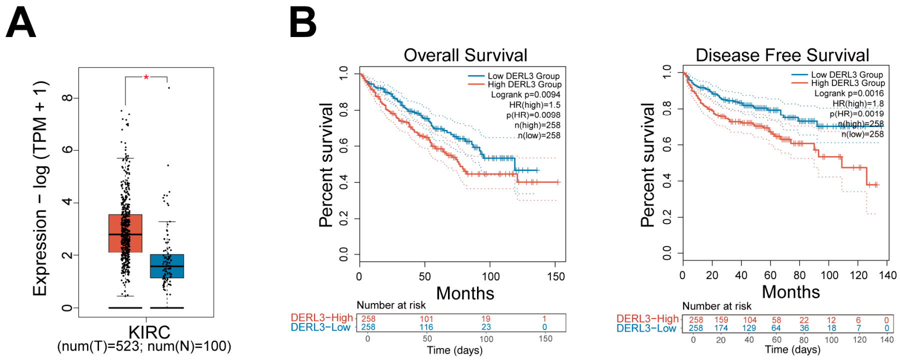

Figure S2. *Public dataset analysis of DERL3 expression and prognosis in ccRCC.* (**A**) DERL3 expression in ccRCC tumor tissues and normal kidney tissues based on public datasets. DERL3 expression was higher in tumor tissues than in normal controls. (**B**) Kaplan-Meier analyses of disease-free survival and overall survival in the TCGA-KIRC cohort stratified by median DERL3 expression (high, n = 258; low, n = 258). Two-sided log-rank tests were used (overall survival, P = 0.0094; disease-free survival, P = 0.0016). Censoring marks and numbers at risk at the labeled time points are shown below each curve.

Table S1. *siRNA sequences used for candidate resistance gene screening.*

Gene	siRNA	Sense strand，5′→3′	Antisense strand，5′→3′
DERL3	si-DERL3-1	GAUUCAGCUUCUUCUUCAA	UUGAAGAAGAAGCUGAAUC

Gene	siRNA	Sense strand，5′→3′	Antisense strand，5′→3′
DERL3	si-DERL3-2	UGAAGAAGAAGCUGAAUCC	GGAUUCAGCUUCUUCUUCA
ZDHHC2	si-ZDHHC2-1	CAGCAUAAACCCAGGAAAA	UUUUCCUGGGUUUAUGCUG
ZDHHC2	si-ZDHHC2-2	GGAAAACACUGGCGAACAA	UUGUUCGCCAGUGUUUUCC
SNORA33	si-SNORA33-1	CCUCUCAGGAAAGUAGCAA	UUGCUACUUUCCUGAGAGG
SNORA33	si-SNORA33-2	AGCAACUUGGAAUAGAAUC	GAUUCUAUUCCAAGUUGCU
NPRL2	si-NPRL2-1	CUGUCCUGUGUGCAUCGAA	UUCGAUGCACACAGGACAG
NPRL2	si-NPRL2-2	AUGAGUCCAACACCAUCCA	UGGAUGGUGUUGGACUCAU
TRIB3	si-TRIB3-1	GGUGUACCCCGUCCAGGA	UUCCUGGACGGGGUACACC
TRIB3	si-TRIB3-2	GGACCUGAGAUACUCAGCU	AGCUGAGUAUCUCAGGUCC
TRIB3	si-TRIB3-3	GAUGAUUCCCUGUGGGACA	UGUCCCACAGGGAAUCAUC
CD276/B7-H3	si-CD276-1	UGAAACACUCUGACAGCAA	UUGCUGUCAGAGUGUUUCA
CD276/B7-H3	si-CD276-2	AAAGAAGAUGAUGGACAAG	CUUGUCCAUCAUCUUCUUU

Table S2. *Primer sequences used for qRT-PCR.*

Gene	Forward primer, 5′–3′	Reverse primer, 5′–3′
DERL3	CTCACTTTCCAGGCACCGT	TAGTAGATATGGCCCACCGC
GAPDH	GTCTCCTCTGACTTCAACAGCG	ACCACCCTGTTGCTGTAGCCAA
